# Plankton Microorganisms Coinciding with Two Consecutive Mass Fish Kills in a Newly Reconstructed Lake

**DOI:** 10.1100/2012/504135

**Published:** 2012-05-01

**Authors:** Andreas Oikonomou, Matina Katsiapi, Hera Karayanni, Maria Moustaka-Gouni, Konstantinos Ar. Kormas

**Affiliations:** ^1^Department of Ichthyology and Aquatic Environment, School of Agricultural Sciences, University of Thessaly, 384 46 Volos, Greece; ^2^Department of Botany, School of Biology, Aristotle University of Thessaloniki, 541 24 Thessaloniki, Greece; ^3^Department of Biological Applications and Technology, University of Ioannina, 451 10 Ioannina, Greece

## Abstract

Lake Karla, Greece, was dried up in 1962 and its refilling started in 2009. We examined the Cyanobacteria and unicellular eukaryotes found during two fish kill incidents, in March and April 2010, in order to detect possible causative agents. Both microscopic and molecular (16S/18S rRNA gene diversity) identification were applied. Potentially toxic Cyanobacteria included representatives of the *Planktothrix* and *Anabaena* groups. Known toxic eukaryotes or parasites related to fish kill events were *Prymnesium parvum* and *Pfiesteria* cf. *piscicida*, the latter being reported in an inland lake for the second time. Other potentially harmful microorganisms, for fish and other aquatic life, included representatives of Fungi, Mesomycetozoa, Alveolata, and Heterokontophyta (stramenopiles). In addition, Euglenophyta, Chlorophyta, and diatoms were represented by species indicative of hypertrophic conditions. The pioneers of L. Karla's plankton during the first months of its water refilling process included species that could cause the two observed fish kill events.

## 1. Introduction

Planktonic Cyanobacteria and unicellular eukaryotes belonging to different functional groups constitute key components of aquatic ecosystems [1]. Among the unicellular plankton there are species that negatively influence the ecosystem [2, 3]. Several of these microorganisms lack distinct morphological features. Even if taxonomically useful morphological features are present, they may get lost throughout sampling, preservation, and examination procedures [4] making identification by traditional microscopic methods difficult. Molecular techniques have spawned new ways to access the diversity of the microbial world. Yet, molecular techniques have limitations [5]. Therefore, a combination of molecular techniques and microscopy methods is required in order to uncover the diversity of the microbial world [6].

Mass fish kills are known to occur in eutrophic lakes. They have been attributed mostly to hypoxic/anoxic conditions or uncommonly high/low temperatures. Other factors, related or not to the eutrophication, include floods, droughts, cyclonic storms, habitat loss, low water flow, and abrupt water level fluctuations [7]. Due to the changes of the grazing pressure, fish kills may lead to considerable changes in the food web structure of the lake ecosystem, with diminishing consequences for the possibilities of using the lake for recreation, fishing, or as a source of drinking water. Although such mass mortality events are well documented in the literature, to the best of our knowledge, there is no such data on newly reconstructed lakes.

In freshwater, the haptophyte *Prymnesium parvum* is considered one of the most dangerous microorganisms and is responsible for adverse effects on aquatic organisms [8] and in particular for several fish kill incidents [9]. It poses a serious threat to several ecosystems since it survives in a wide range of salinities and blooms in coastal and brackish inland waters worldwide [10, 11]. In Lake Koronia, Greece, *P. parvum *coincided with a mass death of birds and fish [2, 12]. The dinoflagellate *Pfiesteria* species can harm fish in coastal waters [13, 14] and has caused fish kills under certain circumstances in North Carolina, USA [13]. No *Pfiesteria*-induced fish kills have ever been reported in Mediterranean coastal waters, while the only, and most unusual, inland ecosystem where *Pfiesteria* has been reported is Ace Lake, Antarctica [15].

While acute fish kills due to toxic algae are well studied, another less obvious impact of toxic/parasitic unicellular eukaryotes is that exposure of aquatic animals to their toxins or parasitism might induce serious sublethal effects, including predisposing these populations to various infectious diseases resulting in, for example, reduction of growth and reproduction [8, 16]. This situation might be even more severe if one considers that we know only a few of the toxic/parasitic eukaryotes that can cause fish kills, while on the other hand our concept on the existing species diversity of the microscopic eukaryotes is still expanding [17]. This led us to investigate the planktonic Cyanobacteria and microeukaryotes of a newly reconstructed lake (Lake Karla, central Greece) during two consecutive fish kill events which occurred in less than six weeks. The aims of this study were to supplement the limited knowledge on the plankton Cyanobacterial and microeukaryotic diversity of newly reconstructed lakes and to identify potentially toxin-producing and parasitic taxa which coincided with the fish kill events and might have deleterious effects on the ecosystem.

## 2. Materials and Methods

### 2.1. Study Area

**Figure 1:**
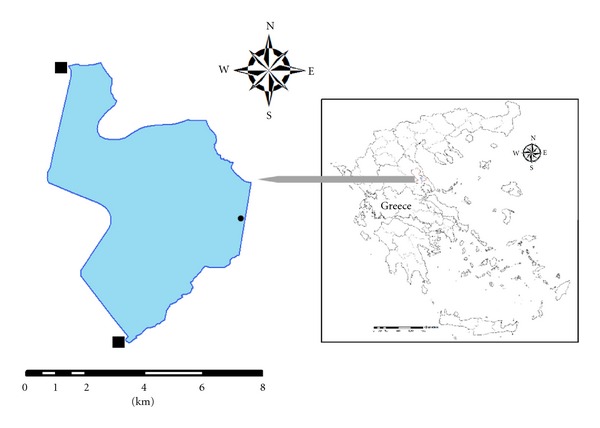
Map of Lake Karla, Greece, and sampling point (black dot). Black squares show points of inflowing water for reconstruction purposes. Centre of the lake is at 39°29′00′′ N, 22°49′00′′ E.

Lake Karla (Figure 1) is located in central Greece (39°29′02′′ N, 22°51′41′′ E). It formerly covered an area of ca. 180 km^2^ but in the beginning of the 1960s it was drained through a tunnel leading the lake's drainage to the nearby Pagasitikos Gulf. A small permanent marsh remained at the area that once covered the lake. The structure and function of L. Karla was correlated with River Pinios, as the flooding events of the river supplied the lake with water rich in nutrients [18]. Several biological and physical-chemical criteria characterized the lake as a eutrophic but with high stability before its drainage [19]. It was not until the 1990s that the refilling of the lake was decided by inflowing water from the nearby River Pinios. Its actual filling started in September 2009, after building a peripheral dam which covers 38 km^2^. We sampled in L. Karla in March and April 2010, during two fish kill events. As reported in local newspapers, the dead fish floated in the lake and lined along the shores of a 3.5 to 5 km stretch.

Water samples for microscopic analysis were collected on 17 March and 20 April 2010 at ca. 0.5 m depth from the water level pier at the southeast end of the lake (Figure 1). Three replicates of 500 mL each were collected in polyethylene bottles. Two of them were fixed with Lugol's solution and formaldehyde, while one was retained fresh for direct microscopic analysis. Water temperature, dissolved oxygen, salinity, and pH were measured in situ using a WTW sensor (Weilheim, Germany).

For each sampling date, at least three replicates of live and preserved samples were examined in sedimentation chambers using an inverted microscope with phase contrast (Nikon SE 2000). Cyanobacteria and microscopic eukaryotes were identified using classical taxonomic keys and previous works [20–23]. Phytoplankton counts (cells, colonies, and coenobia) were performed using the Utermöhl's sedimentation method [24]. For biomass (mg L^−1^) estimation, the dimensions of 30 individuals (cells, filaments, or colonies) of each species were measured using tools of a digital microscope camera (Nikon DS-L1), while mean cell or filament volume estimates were calculated using appropriate geometric formulae, as described previously [25, 26]. Species and taxonomical groups comprising more than 10% (w/w) of the total phytoplankton biomass were considered to be dominant.

Water samples for DNA extraction were transported to the laboratory in 4-L collapsible plastic bottles (Nalgene, Rochester NY, USA) and processed within 1 h of collection. After screening through a 180 *μ*m mesh net to exclude larger eukaryotes and particles, 200–250 mL of water was filtered through a 0.2 *μ*m pore size Polycarbonate Isopore filter (Sartorius, Goettingen, Germany). The filtration was conducted under reduced pressure (≤100 mmHg) to prevent cell damage. Filters were stored immediately at −80°C until further analysis.

DNA was extracted using the UltraClean Soil DNA isolation kit (MoBio Laboratories, Carlsbad CA, USA) according to the manufacturer's protocol after slicing the filter with a sterile scalpel. The concentration of bulk DNA was estimated by spectrophotometry (NanoDrop ND-1000, NanoDrop Technologies, Wilmington DE, USA) and ranged between 11.9 and 15.4 ng *μ*L^−1^ for the March and April samples, respectively. For PCR amplification, approximately 12 ng of environmental DNA was used as template for both samples. The 18S rRNA gene was amplified using the eukaryote specific primers EukA (5′-AACCTGGTTGATCCTGCCAGT-3′) and EukB (5′-GATCCTTCTGCAGGTTCACCTAC-3′) [27] for the March sample, while the primers EukA and Euk1633rE (5′-GGGCGGTGTGTACAARGRG-3′) [28] were used for amplification of the 18S rRNA gene for April sample.

PCR for the amplification of the March sample included an initial denaturation step at 95°C for 15 min, which was followed by 40 cycles consisting of denaturation at 95°C for 45 s, annealing at 55°C for 1 min, and elongation at 72°C for 2 min and 30 s; a final 7 min elongation step at 72°C was included. The PCR protocol for the April sample included an initial denaturation step at 95°C for 2 min followed by 40 cycles of denaturating at 95°C for 40 s, annealing at 50°C for 40 sec, and elongation at 72°C for 2 min and 15 s, with an additional step of final elongation at 72°C for 1 min. Each PCR from the two samples was repeated with different cycle numbers (between 20 and 37). The lowest number of cycles that gave a positive signal, that is, 26 and 28 cycles for the March and April sample, respectively, was further used in order to eliminate some of the major PCR innate limitations [29, 30] and to avoid differential representation of 18S rRNA genes with low and high copy numbers.

For PCR amplification of the Cyanobacterial 16S rDNAs, we used the Cyanobacteria-specific primers CYA106f (5′-CGGACGGGTGAGTAACGCGTGA-3′), CYA781r(a) (5′-GACTACTGGGGTATCTAATCCCATT-3′), and CYA781r(b) (5′-GACTACAGGGGTATCTAATCCCTTT-3′) [31]. PCR included an initial denaturation step at 94°C for 5 min, which was followed by 40 cycles consisting of denaturation at 94°C for 30 s, annealing at 57°C for 30 s, and elongation at 72°C for 3; a final 5 min elongation step at 72°C was included. Cycle optimization was performed as above which resulted in 26 cycles for the March sample. In April 2010, no sample was analysed for 16S rRNA gene diversity since the vast majority of the observed morphospecies was observed microscopically.

The PCR products from both the Eukarya- and Cyanobacteria-specific amplifications were visualized on a 1% agarose gel under UV light, purified using the Montage purification kit (Millipore Inc, Molsheim, France). The purified PCR products were ligated into the PCR XL TOPO Vector (Invitrogen-Life Technologies, Carlsbad CA, USA) and transformed in electrocompetent *Escherichia coli* cells according to the manufacturer's specifications. For each clone library a maximum of 151 clones were sequenced, each containing an insert of ca. 1800/1600 or 680 bp for the Eukarya and Cyanobacteria, respectively. These clones were grown in liquid Luria-Bertani medium with kanamycin and their plasmids were purified using the Nucleospin Plasmid QuickPure kit (Macherey-Nagel GmbH and Co. KG, Düren, Germany) for DNA sequencing. Sequence data were obtained by capillary electrophoresis (Macrogen Inc., Seoul, Korea) using the BigDye Terminator kit (Applied Biosystems-Life Technologies, Carlsbad, CA, USA) with the set of primers M13F (5′-GTAAAACGACGGCCAG-3′) and M13R (5′-CAGGAAACAGCTATGAC-3′). For the eukaryotic clones, intermediate sequencing was performed using the primer 1179rE (5′-CCCGTGTTGAGTCAAATT-3′) [32]. Each sequence read was approximately 850 bp. For each individual clone, forward, reverse, and intermediate—for the Eukarya—reads were assembled, and then the assembled sequences were checked for chimeras. The Pintail program (http://www.bioinformatics-toolkit.org/Web-Pintail/, [33]) was used for the detection of putative chimeric sequences. Chimeras were discarded from the dataset. Using the multiple alignment program CLUSTALW2 (http://www.ebi.ac.uk/Tools/clustalw2/index.html/) and based on 98% gene similarity as a phylotype cutoff [17, 34], clones were grouped together and considered members of the same phylotype. All sequences were compared with the BLAST function (http://www.ncbi.nlm.nih.gov/BLAST/) for the detection of closest relatives. Sequence data were compiled using the MEGA4 software [35] and aligned with sequences obtained from the GenBank (http://www.ncbi.nlm.nih.gov/) database, using the ClustalX aligning utility. Phylogenetic analyses were performed using the MEGA version 4 software [35] and the topology of the tree was based on neighbour-joining according to Jukes-Cantor. Bootstrapping under parsimony criteria was performed with 1,000 replicates. Sequences of unique phylotypes found in this study have GenBank accession numbers JN090861-JN090912 and JN090913-JN090923 for the eukaryotes and Cyanobacteria, respectively.

Library clone coverage was calculated by the formula of the Good's C estimator [1 − (*n*_*i*_/*N*)] [36], where *n*_*i*_ is the number of phylotypes represented by only one clone and *N* is the total number of clones examined in each library. The number of predicted phylotypes for each clone library was estimated after the abundance-based richness formula *S*_Chao1_ [37, 38].

## 3. Results and Discussion

We investigated the composition of plankton Cyanobacteria and unicellular eukaryotes by combing molecular, 18S/16S rRNA gene diversity, and microscopic analysis in Lake Karla during two fish kill events which happened within the first year of the lake's partial reconstruction. The prevailing abiotic factors (Table 1) indicated that dissolved oxygen (5.6–5.8 mg L^−1^) was not limited, while the elevated salinity (7.6–8.1 psu) was possibly attributed to the drainage of the previous lake as well as the result of intensive agricultural and livestock use for four decades. Irrigation in the absence of leaching can increase soil salinity [39] and continued application of livestock manure to agricultural land may result in an accumulation of salt in soil [40].

**Figure 2:**
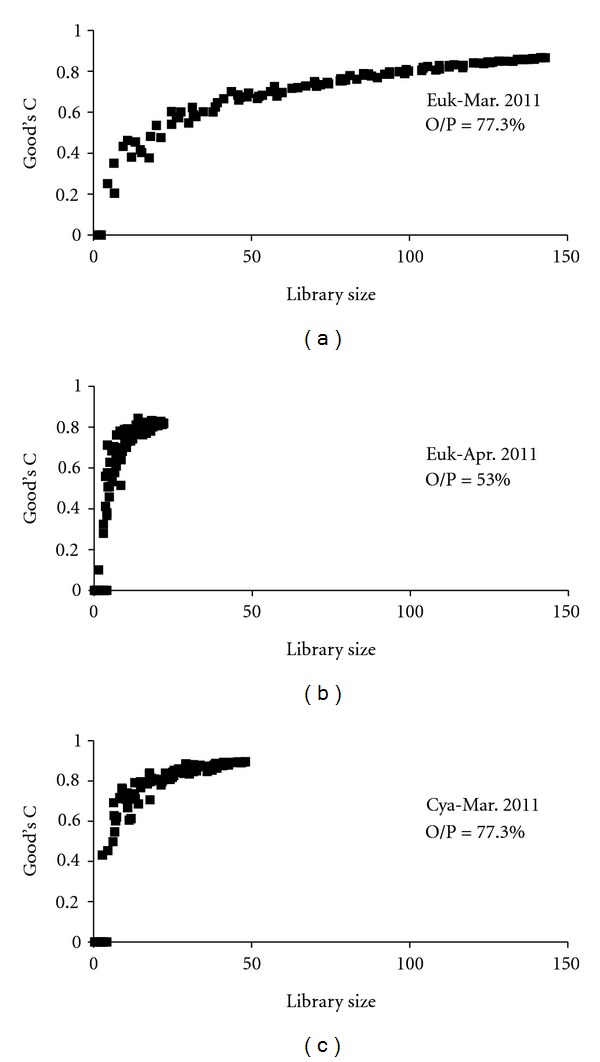
rRNA gene clone library coverage based on Good's C estimator of the unicellular eukaryotes (Euk) and Cyanobacteria (Cya) from Lake Karla, Greece. O/P = ratio of observed-to-predicted number of phylotypes.

**Figure 3:**
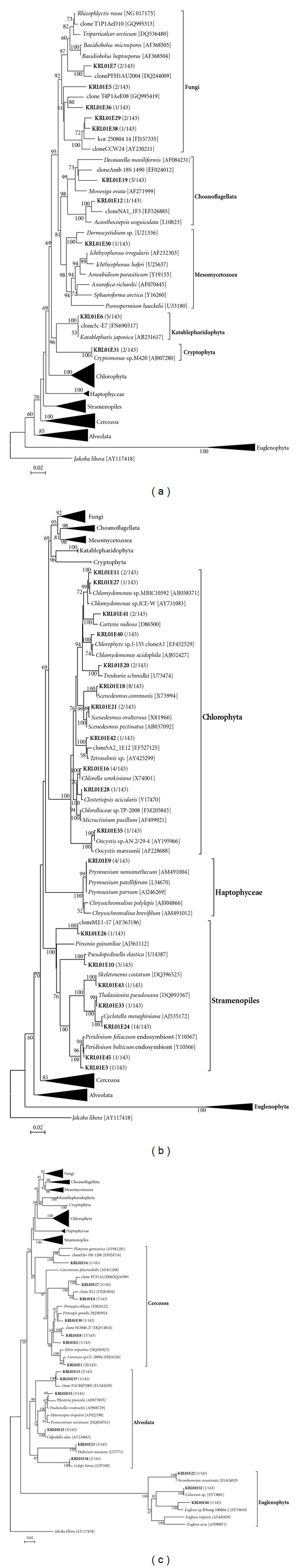
(a) Phylogenetic tree of relationships of 18S rDNA (ca. 1800 bp) of the representative unique (grouped on ≥98% similarity) eukaryotic clones (in bold) of the taxa Fungi, Choanoflagellata, Mesomycetozoea, Katablepharidophyta, and Cryptophyta, found in the Lake Karla water column, March 2010, based on the neighbour-joining method as determined by distance Jukes-Cantor analysis. One thousand bootstrap analyses (distance) were conducted. GenBank numbers are shown in parentheses. Numbers in parentheses indicate the relative abundance in the clone library. Scale bar represents 2% estimated. (b) Phylogenetic tree of relationships of 18S rDNA (ca. 1800 bp) of the representative unique (grouped on ≥98% similarity) eukaryotic clones (in bold) of the taxa Chlorophyta, Haptophyta, and Heterokontophyta (stramenopiles), found in the Lake Karla water column, March 2010, based on the neighbour-joining method as determined by distance Jukes-Cantor analysis. One thousand bootstrap analyses (distance) were conducted. GenBank numbers are shown in parentheses. Numbers in parentheses indicate the relative abundance in the clone library. Scale bar represents 2% estimated. (c) Phylogenetic tree of relationships of 18S rDNA (ca. 1800 bp) of the representative unique (grouped on ≥98% similarity) eukaryotic clones (in bold) of the taxa Cercozoa, Alveolata and Euglenophyta, found in the Lake Karla water column, March 2010, based on the neighbour-joining method as determined by distance Jukes-Cantor analysis. One thousand bootstrap analyses (distance) were conducted. GenBank numbers are shown in parentheses. Numbers in parentheses indicate the relative abundance in the clone library. Scale bar represents 2% estimated.

**Figure 4:**
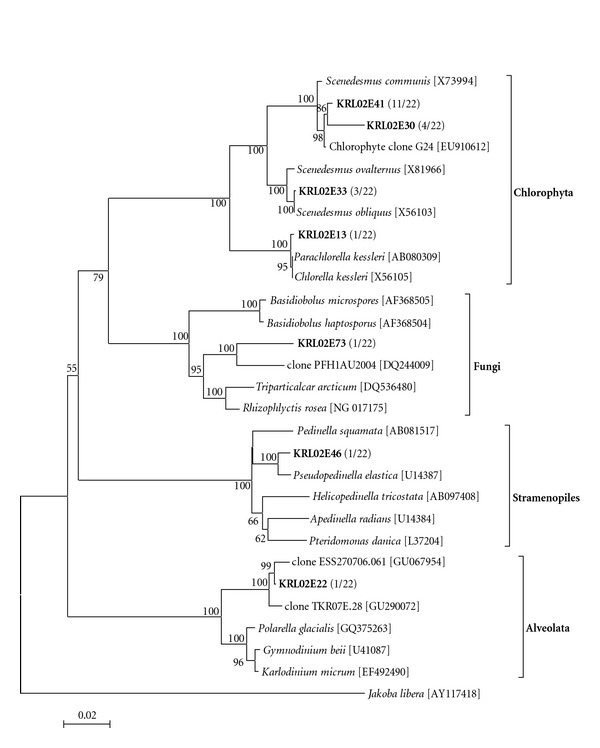
Phylogenetic tree of relationships of 18S rDNA (ca. 1600 bp) of the representative unique (grouped on ≥98% similarity) eukaryotic clones (in bold) found in the Lake Karla water column, April 2010, based on the neighbour-joining method as determined by distance Jukes-Cantor analysis. One thousand bootstrap analyses (distance) were conducted. GenBank numbers are shown in parentheses. Numbers in parentheses indicate the relative abundance in the clone library. Scale bar represents 2% estimated.

The two eukaryotic clone libraries revealed that 45 phylotypes occurred in March and only seven in April 2010. However, in both cases, rarefaction curves (Figure 2) reached saturation levels for both clone libraries according to the Good's C estimator, indicating that the majority of the existing phylotypes were revealed. Based on the 18S rRNA gene diversity (Figures 3 and 4), members of the Chlorophyta, Cercozoa, Heterokontophyta (stramenopiles), Alveolata, Fungi, Euglenophyta, Choanoflagellata, Haptophyta, Mesomycetozoea, Katablepharidophyta, and Cryptophyta (Figures 2 and 3) were found. Chlorophyta was the most phylotype-rich group in both samplings, while the next most abundant phylotypes belonged to the Cercozoa, Alveolata, and stramenopiles.

**Figure 5:**
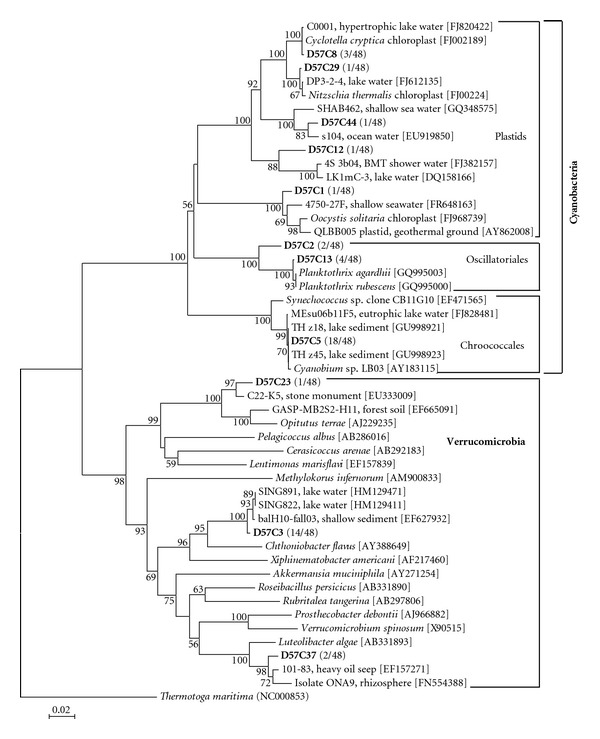
Phylogenetic tree of relationships of 16S rDNA (ca. 660 bp) of the representative unique (grouped on ≥98% similarity) Cyanobacterial clones (in bold), March 2010, based on the neighbour-joining method as determined by distance Jukes-Cantor analysis. One thousand bootstrap analyses (distance) were conducted. GenBank numbers are shown in parentheses. Numbers in parentheses indicate the relative abundance in the clone library. Scale bar represents 2% estimated.

The Cyanobacteria 16S rRNA gene clone library coverage was satisfactory (Figure 2) and showed (Figure 5) that Cyanobacteria were represented by phylotypes related to the *Planktothrix* group, the Chroococcales, and several algal plastids. Along with these phylotypes, three Verrucomicrobia-like phylotypes were also retrieved, reinforcing the notion that some Verrucomicrobia are associated with Cyanobacteria-dominated waters [41, 42].

**Figure 6:**
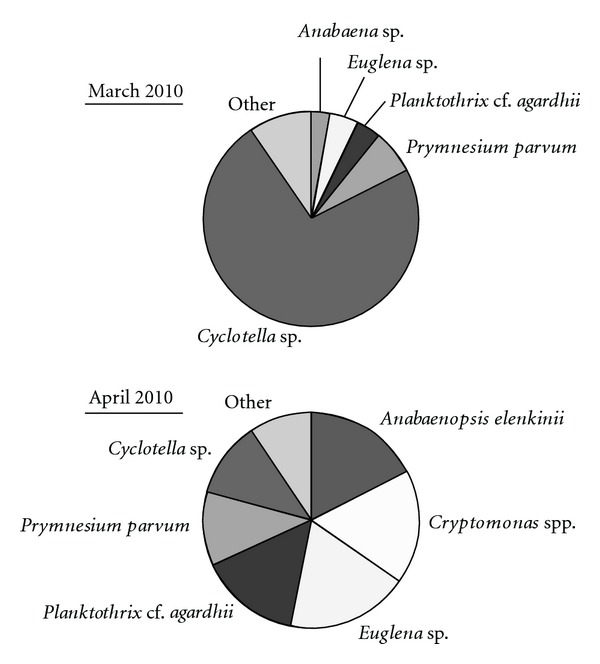
Relative biomass of the major taxa (90% dominance) recognized with light microscopy in the Lake Karla water column.

Microscopic analysis (Figure 6) of phytoplankton gave a slightly different picture of the phytoplankton dominance. In March 2010, the diatom *Cyclotella* sp. dominated followed by *Prymnesium parvum *(Haptophyta), *Planktothrix* cf. *agardhii* (Cyanobacteria), *Euglena* sp. (Euglenophyta) and *Anabaena* sp. (Cyanobacteria) and from Alveolata *Pfiesteria* cf. *piscicida* (the latter consisted 0.4% of the high 46.5 mg L^−1^ total biomass and for this it is not included in Figure 6). Most of these microorganisms have been also found in April 2010 but in lower biomass. Nevertheless, the phylotypes of these organisms have been found in the respective clone libraries from both dates.

The slight discrepancy between the two approaches is expected (e.g., [43]) as PCR-based phylotype abundance is not quantitative but rather shows relative differences and can also be biased towards some groups. On the other hand, microscopic identification of unicellular phytoplankton can be problematic for some organisms, especially for these with complex/uncertain life cycles (e.g., [3, 25]). Thus, both approaches provide complementary rather redundant information. The gains of using both methods have already been depicted in limnological analysis (e.g., [42]) and especially for the unicellular eukaryotes [4, 6, 43].

The occurrence of diverse Chlorophyta phylotypes in both samplings (Figures 3 and 4), most of which were affiliated with well-characterized species, is related to the hypertrophic conditions prevailing in L. Karla. Chlorophyta are indicative of ecosystems receiving high nutrient loadings [1]. They have been found to dominate the clone library of a hypertrophic, polluted and heavily modified lake in Greece [3]. Some of these phylotypes, for example, *Scenedesmus *species, may constitute an important fraction of the freshwater total phytoplankton biomass, particularly in nutrient-rich ecosystems [44]. *Scenedesmus *species have capabilities of successful air dispersal and colonization of new aquatic habitats [45]. The hypertrophic conditions of the newly reconstructed L. Karla render its future rather erratic, since the prediction of community and ecosystem dynamics is decreased in eutrophic systems [46].

Apart from the Chlorophyta, other microorganisms in this study are associated with eutrophic/hypertrophic conditions. The found Euglenophyta-related phylotypes (Figure 3(c)) were affiliated with the genera *Colacium*, *Euglena* and *Strombomonas.* Members of the Euglenophyta are known to be abundant in highly eutrophic environments and on sediments polluted with organic matter [47]. Euglenophyta are considered biological indicators of organic pollution in seawater [48]. Cryptophyta (Figure 3(a)) are also a group forming blooms in eutrophic environments, yet their abundance are low due to high grazing rates of their protozoan predators [49]. Katablepharidophyta (Figure 3(a)) which were formerly classified as a subgroup of Cryptophyta, are now considered to be a sister group of Cryptophyta [50] and could have similar environmental preferences. Choanoflagellata (Figure 3(a)) are epiphytic microorganisms depending on the quality of available organic matter, and many members of this group are adapted to using dissolved organic matter and colloidal organic particles [51].

The Cercozoa-related phylotypes (Figure 3(c)) were related to uncultivated environmental clones. Some well-characterized species such as *Ebria tripartita, Cercomonas plasmodialis*, and species of the genus *Protaspis* were affiliated with our retrieved sequences and fell in the Cercozoa taxonomic group. These taxa were also identified microscopically. Phylotypes KRL01E17 and KRL01E4 formed a novel clade in the Cercozoa, highly supported by the bootstrap test. Cercozoa phylotypes have been recovered from many different environments [52] but most of them are defined by molecular data and display huge morphological and ecological diversity [53]. They are mainly heterotrophs, including bacterivorous and predaceous species that phagocytize the cytoplasm of diatoms in marine ecosystems [54]. Cyst formation is a widespread characteristic among the Cercozoa [55], which probably allows their presence in anoxic sediments [56]. Members of the genus *Protaspis*, which was also recognized microscopically, comprise common predators in benthic marine ecosystems [55].

It is difficult to infer the trophic role of an organism by its phylogenetic position; however, the fact that most of the prementioned species/taxonomic groups have been detected with light microscopy of fixed and fresh samples in high numbers enforces the notion that these microorganisms are metabolically active in L. Karla. Based on the basic principle of ecology that the function of an ecosystem is defined by its dominant taxa, it is reasonable to characterize L. Karla on the basis of its plankton as a hypertrophic system. Such systems tend to host various parasites as well as known toxin producers. Increased nutrient loadings are known to be associated with outbreaks of microparasitic species and blooms of harmful microalgae can also be indirectly promoted by nutrients inputs [57]. In the current study, such harmful eukaryotes belonging to the Alveolata, Fungi, Mesomycetozoea, and Haptophyta (Figures 3 and 4) along with some toxin-producing Cyanobacteria (Figure 5), have been identified by both molecular and microscopic analysis representing a very interesting but not previously described taxonomic and functional association [58].

Strict parasites are grouped in the Alveolata (Figures 3(c) and 4), as suggested by [59]. *Colpodella edax* can parasitize on Chlorophyta or Cryptophyta and can predate on protozoans smaller in size sucking out their cell contents by means of a rostrum [60]. Reference [59] associated this trophic strategy (myzocytosis) with parasitism. The Fungi (Figures 3(a) and 4) are exclusively composed of saprotrophs, known parasites of the phytoplankton community. Members of the Chytridiomycota can regulate the population of diatoms [59, 61]. Infection of certain phytoplankton species may suppress its development, thus Fungi parasitism can be an important factor controlling seasonal succession [61].

The taxonomic group of Mesomycetozoea (Figure 3(a)) includes facultative or obligate parasites [62]. Two orders have been described in Mesomycetozoea whereof Dermocystida consists exclusively of pathogenic microorganisms infecting fish (*Dermocystidium* sp.) as well as mammals and birds [62]. Members of this group have been found in another degraded lake ecosystem [3].

Known toxin producers such as *Prymnesium parvum* (Haptophyta) and *Pfiesteria* cf. *piscicida* (Alveolata) were also observed both in the clone libraries and by microscopic observations (Figures 3(b) and 6). To the best of our knowledge, it is the first time that these species occur simultaneously in the same ecosystem. *P. parvum* may form extensive blooms with major biogeochemical and ecological impact in brackish or inland waters [9, 63]. Massive kills of fish and birds have been attributed to blooms of *Prymnesium* [9, 25, 64]. *Pfiesteria piscicida* and *P. vonstochii* are parasites with similar feeding strategy and life cycle [65]. Temperature and salinity were suitable for the presence of *Pfiesteria* cf. *piscicida* in the lake as the species is detected in salinity ranging from 0.1–17.8 psu and temperature ranging from 3.2 to 25.5°C [66]. Toxin production of the *Pfiesteria* species increases in high nutrient loadings [13, 67, 68]. The genus *Peridinium* belonging to Alveolata (Figures 3(b) and 4) also includes species apparently related with toxin production [69]. Finally, the harmful organisms community of L. Karla hosts well-known toxin-producing Cyanobacteria [70] like *Planktothrix* cf. *agardhii*, *Anabaena* sp., and *Anabaenopsis elenkinii* (Figures 5 and 6).

During our samplings, salinity of L. Karla was elevated, generating the hypothesis that in L. Karla the occurrence of brackish or marine protists is feasible. Indeed, in both samplings we found phylotypes that were closely related to marine stramenopiles [71, 72]. *Cyclotella meneghiniana* present in the clone library of March, which was found by microscopy dominant in March 2010 and was identified as *Cyclotella* sp., is a common diatom species but tends to become abundant in organic, inorganic, heavy metal, or toxin-polluted environments [73]. *C. meneghiniana* has been recorded as being predominant or in remarkable occurrences in five polluted rivers and in four hypertrophic lakes [73]. *Thalassiosira* genus constitutes primarily of marine species (about 180 described species), while at least 12 species have been observed in freshwater ecosystems [74, 75]. The genus *Skeletonema* significantly contributes to phytoplankton blooms in many regions (e.g., [76–78]). In particular, *Skeletonema costatum* is a species that flourishes in nutrient-rich coastal waters throughout the world [79].

The presence of marine species within the stramenopiles (Figure 3(b)) poses the issue of the origin of these species in our study site. Karla is a newly reconstructed lake which is still under constant change and new microscopic eukaryotes colonize that ecosystem. Cyst formation is known for most of the groups observed like Cercozoa [54], Haptophyta [80], and Alveolata [81], so some microorganisms could have remained in the marsh and in the soil that formerly was the lakebed. The origin of the dominant freshwater microscopic eukaryotes (*C. meneghiniana*, *Scenedesmus* species) can be attributed to the inflow of River Pinios (the species were observed in the River's plankton, Moustaka-Gouni et al. unpublished data). Air dispersal is another possible vector for microorganisms. Chlorophyta have been found to be dominant in aerobiological studies [82] and are successful colonists in new aquatic habitats [45, 83].

In conclusion, our study showed that during two consecutive fish kill incidents which occurred in the recently reconstructed Lake Karla, Greece, in a six-week interval, the lake's water represented a cocktail of potentially toxic, *Planktothrix* cf. *agardhii*, *Prymnesium parvum,* and *Pfiesteria* cf. *piscicida *and parasitic species including *Dermocystidium* sp. Since the water temperature was far from the freezing point and the dissolved oxygen concentration was not even close to hypoxia, it is possible that the fish kills were caused by some of the microorganisms we observed. Apart from this risk, another problem for the ecosystem during the filling process of Lake Karla is the occurrence of other plankton, both freshwater and marine species, which are typical of eutrophic-hypertrophic conditions.

## Figures and Tables

**Table 1:** Prevailing physical and chemical parameters in L. Karla.

	Temperature (°C)	Salinity (PSU)	Dissolved oxygen (mg/L)	pH
17/03/2010	15.6	7.6	5.8	8.3
20/04/2010	17.2	8.1	5.6	8.0
